# Pilot-scale genome-wide association mapping in diverse sorghum germplasms identified novel genetic loci linked to major agronomic, root and stomatal traits

**DOI:** 10.1038/s41598-023-48758-2

**Published:** 2023-12-08

**Authors:** Ajay Prasanth Ramalingam, Williams Mohanavel, Rohit Kambale, Veera Ranjani Rajagopalan, Sandeep R. Marla, P. V. Vara Prasad, Raveendran Muthurajan, Ramasamy Perumal

**Affiliations:** 1 Tamil Nadu Agricultural University, Coimbatore, India; 2 Department of Agronomy, Kansas State University, Manhattan, KS USA; 3 Agricultural Research Center, Kansas State University, Hays, KS USA

**Keywords:** Genetics, Plant sciences

## Abstract

This genome-wide association studies (GWAS) used a subset of 96 diverse sorghum accessions, constructed from a large collection of 219 accessions for mining novel genetic loci linked to major agronomic, root morphological and physiological traits. The subset yielded 43,452 high quality single nucleotide polymorphic (SNP) markers exhibiting high allelic diversity. Population stratification showed distinct separation between *caudatum* and *durra* races. Linkage disequilibrium (LD) decay was rapidly declining with increasing physical distance across all chromosomes. The initial 50% LD decay was ~ 5 Kb and background level was within ~ 80 Kb. This study detected 42 significant quantitative trait nucleotide (QTNs) for different traits evaluated using FarmCPU, SUPER and 3VmrMLM which were in proximity with candidate genes related and were co-localized in already reported quantitative trait loci (QTL) and phenotypic variance (R^2^) of these QTNs ranged from 3 to 20%. Haplotype validation of the candidate genes from this study resulted nine genes showing significant phenotypic difference between different haplotypes. Three novel candidate genes associated with agronomic traits were validated including Sobic.001G499000, a potassium channel tetramerization domain protein for plant height, Sobic.010G186600, a nucleoporin-related gene for dry biomass, and Sobic.002G022600 encoding AP2-like ethylene-responsive transcription factor for plant yield. Several other candidate genes were validated and associated with different root and physiological traits including Sobic.005G104100, peroxidase 13-related gene with root length, Sobic.010G043300, homologous to Traes_5BL_8D494D60C, encoding inhibitor of apoptosis with iWUE, and Sobic.010G125500, encoding zinc finger, C3HC4 type domain with Abaxial stomatal density. In this study, 3VmrMLM was more powerful than FarmCPU and SUPER for detecting QTNs and having more breeding value indicating its reliable output for validation. This study justified that the constructed subset of diverse sorghums can be used as a panel for mapping other key traits to accelerate molecular breeding in sorghum.

## Introduction

Cereal crops provide over 75% of human calories and projected increase in the global population necessitates 70–100% increase in cereal food production by 2050^1^. Furthermore, declining availability of required fertile land for expanding under cereal crops and challenges due to changing climatic conditions, make it hard to meet global food demand with prevailing agricultural practices^2^. Due to their adaptation to extreme weather events, requirement of less inputs and possession of high nutritional values and therapeutic clues, climate resilient cereal crops are becoming ideal crops for improving global food security^3,4^. Most of the crop improvement programs in these hardy cereals were focused on easy to phenotype traits, such as grain yield, nutritional contents, plant architecture, disease, and insect resistance. However, breeding for abiotic stress tolerance had limited success due to difficulties in the phenotyping of stress-associated traits viz., root morphology, stomatal traits, and gas exchange parameters.

Sorghum [*Sorghum bicolor* (L.) Moench] is the world's fifth most important cereal crop in terms of total production and acreage with 40.25 million ha under cultivation and 58.7 million MT of grain production^5^. Sorghum is grown for a variety of purposes in areas with low or erratic precipitation, including grain for food or livestock feed, forage biomass for livestock feed, and sweet sorghum for syrup and biofuel production^6^. Sorghum exhibits high genetic diversity where ~ 236,617 germplasm accessions including cultivated and weedy relatives are conserved in gene banks around the world^7^. However, utilization of these large germplasm collections in crop improvement program is extremely limited. It is primarily due to the photosensitivity, and non-availability of reliable data on the genetic variation for complex traits of economic interest as well as other factors such as linkage load of undesirable genes, restricted germplasm access, and international exchange regulations^8^. Given the availability of pan-genomes and genetic resources for sorghum improvement^7,9^, it is important to investigate and understand the genetic basis for different complex traits like root architecture and physiological traits that serve as the foundation for improving abiotic stress tolerance in sorghum, particularly drought stress.

Root traits are critical for water and nutrient uptake, climate resilience and productivity, but receive little attention due to phenotyping difficulties. Root system architecture (RSA), determined by primary and lateral root length, number, spread, and biomass, which exhibits greater plasticity in response to environmental shifts, could be critical in developing drought hardy genotypes. Understanding the genetic and molecular basis of RSA may help to improve resource use efficiency and to break any linkage between yield and stress tolerance as in the case of rice^10^. RSA provides the basis for stress tolerance, have been studied extensively to dissect its genetic basis in *Arabidopsis*^11–13^ while limited studies have been attempted in sorghum^14,15^.

Stomatal traits play an important role in regulating crop productivity and stress tolerance under diverse environmental conditions^16^. Stomata plays a vital role in determining transpiration rate (TR) and thus it holds a promising role in maintaining water balance, turgor pressure, nutrient uptake and tolerance to drought and high temperatures. Genetic manipulation of TR and water use efficiency (WUE) has been demonstrated by altering stomatal development, and density in various monocots and dicots^17^. Decrease in stomatal density was found to increase drought tolerance and intrinsic WUE (iWUE) without affecting the yield in cereals^18^. Despite of its greater role in maintaining water balance in plants, only limited studies have been attempted to unravel genetic loci governing stomatal traits in sorghum^18,19^. The majority of genome wide association studies (GWAS) presented in sorghum were conducted with sorghum conversion program germplasm, which limited its success in effectively dissecting genetic loci affecting the traits of interest^20,21^.

Bi-parental mapping populations were commonly used to identify genomic regions underpinning traits of interest in major crops. However, the success rate is limited due to the narrow allelic diversity and low genomic resolution which limited mapping candidate genes underlying traits of interest. With advances in high-throughput genotyping using next generation sequencing (NGS), use of diversified association mapping panels in gene discovery has become popular due to its ability to overcome the significant limitations of bi-parental populations^22^. Several GWAS employ the genotyping-by-sequencing (GBS) strategy to generate dense single-nucleotide polymorphism (SNP) markers covering the whole genome^23^. Several models are available for performing GWAS, from which models like settlement of MLM under progressively exclusive relationship (SUPER) and fixed and random model circulating probability unification (FarmCPU) have shown to be more common models and 3 variance-component multi-locus random-SNP-effect mixed linear model (3VmrMLM) is with high reliable accuracy. The model SUPER is a single locus model which uses bin approach to select associated markers^24^ and the model FarmCPU is a hybrid model which uses both fixed and random effect model decreasing the results with less false positive and negative making it more robust^25^. The model 3VmrMLM have shown to cover quantitative trait nucleotide (QTN)-by-environment interactions (QEIs) and QTN-by-QTN interactions (QQIs) with higher power and accuracy in QTN detection particularly in small mapping population^26^. Although several attempts were made to dissect genetic loci and candidate genes for various agronomic traits through GWAS^20,27–29^, there still remains a gap for understanding the genetic basis of several abiotic stress tolerance related root and physiological traits which are important and critical to make sorghum as a more climate-resilient crop. This study assembled a representative subset of diverse sorghum lines constructed from a larger collection of sorghum germplasm to effectively identify major genetic loci and novel candidate genes for major agronomic, root morphological and physiological traits through GWAS. In addition to identification of significant SNPs, the genetic effect of these SNPs with significant associations to their respective traits were determined from its phenotype in this study for validation. Several studies have validated their SNPs by understanding their genetic effect with the traits’ phenotypes^29–32^. Further, extensive bioinformatics studies were carried out to identify causative gene(s) underlying genetic variation for grain color, plant height and few major physiological traits. Overall, this pilot scale initiative paved way for an extensive GWAS analysis for mapping genetic loci/genes underlying major agronomic and climate resilience traits. The specific objectives were to: (1) construct a representative subset from a large collection of sorghum germplasm accessions using simple sequence repeat (SSR) microsatellite markers, (2) sequence the constructed subset germplasm accessions to understand the allelic diversity and population structure, and (3) perform GWAS using three models (SUPER, FarmCPU and 3VmrMLM) for agronomic, root and physiological traits and identify the novel quantitative trait loci (QTL) and identify candidate genes associated with these traits to validate the study.

## Results

### Population structure and linkage disequilibrium

**Figure 1:**
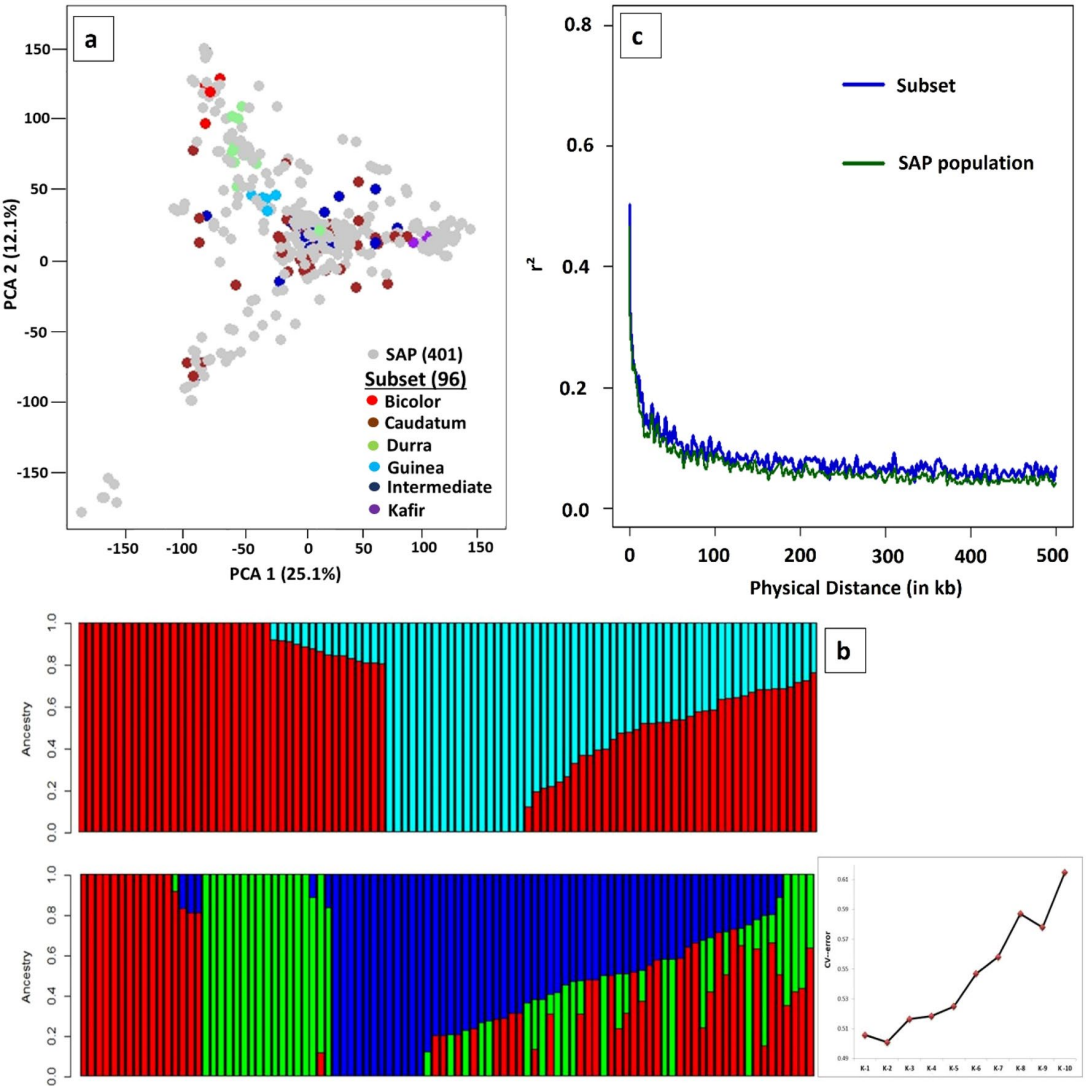
(**a**) Principal component analysis (PCA) of the constructed subset (n = 96) plotted on PCA axis individually and PCA axes built with SAP accessions (n = 401) showing wide diversity and distinct grouping among races. (**b**) **(top).** Population stratification using model-based maximum likelihood approach at K = 2 (optimum K based on CV-error plot) showed a distinct separation of caudatum (red) and durra (light blue) races; (**b**) **(bottom).** Guinea (dark blue) was also distinctly separated along with caudatum (red) and durra (green) races at K = 3 level. (**c**) LD decay of the constructed subset and SAP population indicating decay to the background level (r^2^ < 0.1) within ~ 80 Kb in the constructed subset.

A subset of 96 accessions was constructed as a representative panel from the whole 219 accessions showing distinct grouping of accessions based on their geographical origin (Supplementary Fig. 1). Principal Component Analysis (PCA) of the subset plotted with the axes generated using the sorghum association panel (SAP) showed a cumulative variance of 37.2% using the first two PCs (25.1 and 12.1%), indicating a wide genetic diversity of the subset (Fig. 1a). ADMIXTURE analysis for K = 2 to 10 was performed with tenfold cross-validation (CV), where K = 2 showed the minimum CV error which makes it an optimum number of sub-population in this subset (Fig. 1b). At K = 2, distinct separation was observed between *caudatum* and *durra* with about 60 accessions having ancestry proportion ≥ 0.8 assigned to each sub-population, and at K = 3, distinct separation of guinea race was also observed along with *durra* and *caudatum*. Linkage Disequilibrium (LD) among the filtered SNPs (r^2^) used in the subset were rapidly declining with increasing physical distance for all chromosome, with the initial 50% decay by ~ 5 Kb and decay to the background level (r^2^ < 0.1) within ~ 80 Kb (Fig. 1c). For GWAS, a total of 43,452 high quality SNPs (minor allele frequency (MAF) > 0.05) were filtered exhibiting high regional diversity and distribution in all ten chromosomes covering the sorghum genome (Supplementary Fig. 2).

### Heritability

**Figure 2:**
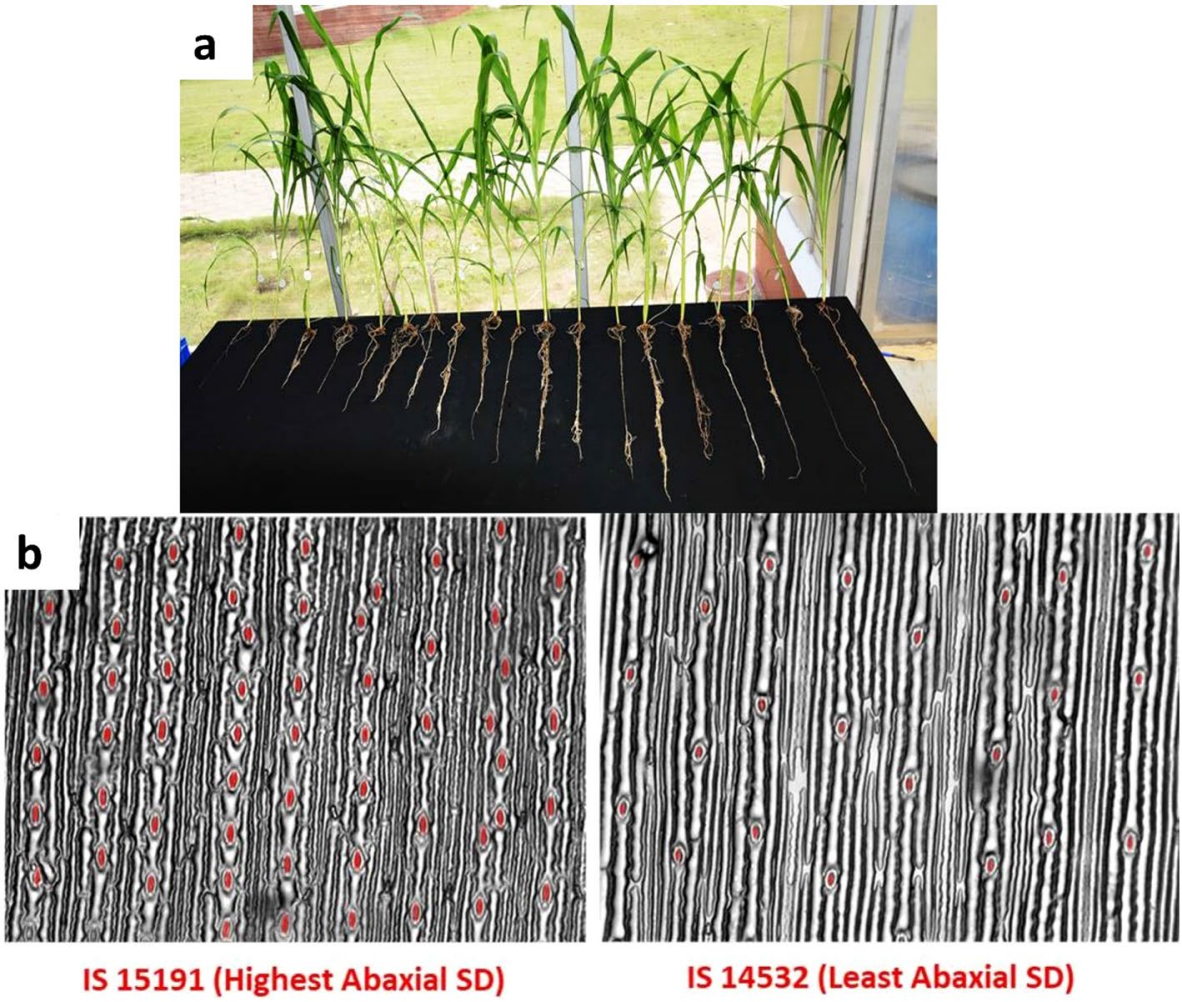
(**a**, **b**) Genetic variation of the root and physiological traits for the constructed subset.

The constructed subset exhibited wide variation for root morphological and physiological traits (Fig. 2) and normal frequency distribution (Supplementary Fig. 3). All the agronomic traits were found to exhibit high broad sense heritability (H^2^) ranging between 0.8 and 0.97. Root morphological and stomatal traits showed medium to high heritability (0.53–0.92). Moderate-to-high heritability exhibited by all the ten traits enabled detection of candidate genes using GWAS (Supplementary Table 1).

### Genome-wide association studies (GWAS)

#### Agronomic traits

**Figure 3:**
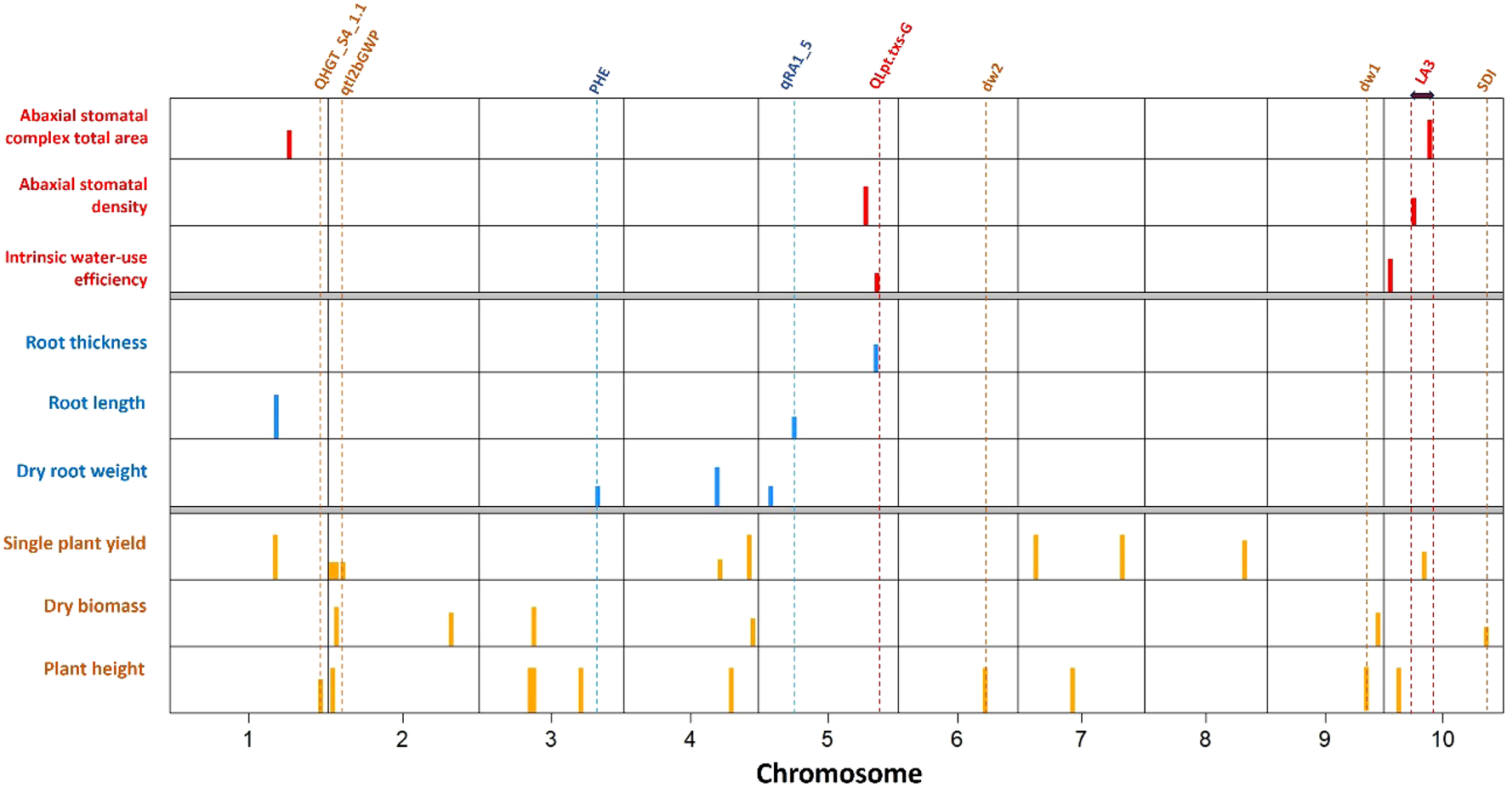
GWAS for all traits on the representative subset using FarmCPU, SUPER and 3VmrMLM methods indicating significant QTNs co-localized in already reported QTL.

The model FarmCPU was able to detect two significant QTNs for dry biomass and six for single plant yield. No significant QTNs were detected by SUPER method for plant height and plant yield. Furthermore, 3VmrMLM method was used, which detected 20 QEIs for all agronomic traits, showing proximity to candidate genes related to plant growth and development. QTNs were also detected by 3VmrMLM from which four QTNs were associated with dry biomass which were same as QEIs detected for dry biomass, no QTNs were detected near candidate genes for plant height and two QTNs were detected to be associated with plant yield (Fig. 3, Supplementary Fig. 4 and Supplementary Table 2).

The method 3VmrMLM detected a non-synonymous QEI (S1_76855452) significantly associated with plant height present in Sobic.001G499000, encoding potassium channel tetramerization domain showing phenotypic variance (R^2^) of 3%. Another non-synonymous QEI (S10_52722710) was found to be significantly associated with dry biomass using 3VmrMLM in Sobic.010G186600, a nucleoporin-related gene showing R^2^ of 3%. Seven QEIs by 3VmrMLM and six QTNs by FarmCPU were found to be significantly associated with single plant yield and present in proximity (within 50 kb) with the candidate genes related to panicle development.

#### Root morphological traits

The methods FarmCPU and SUPER did not detect any significant QTNs for all three root morphological traits (root length, root thickness, dry root weight) whereas, 3VmrMLM detected six significant QTNs (Fig. 3, Supplementary Fig. 5 and Supplementary Table 2). There was no non-synonymous QTNs detected within a candidate gene and all the significant QTNs were in proximity (< 36 kb) with candidate genes related to root growth and development explaining R^2^ ranging from 5 to 19%. A QTN S5_60337757 was significantly associated with root thickness explaining 19% R^2^ and found in proximity with Sobic.005G141801, encoding cadmium-induced protein.

#### Physiological traits

The methods FarmCPU and SUPER did not detect significant QTNs for all other physiological traits evaluated. Whereas, a total of six significant QTNs (iWUE: 2, Abaxial SD: 2; Abaxial SCTA: 2) were detected by using the method 3VmrMLM (Fig. 3, Supplementary Fig. 6 and Supplementary Table 2). A non-synonymous QTN S10_15510030 was detected to be associated with Abaxial SD explaining 20% R^2^ and is present in Sobic.010G125500, encoding zinc finger, C3HC4 type domain. For iWUE, S10_3353158 was detected as a significant QTN present in Sobic.010G043300, which has homologous function in wheat encoding inhibitor of apoptosis 1 showing 11% R^2^.

### Validation of candidate genes-Haplotype analysis

**Figure 4:**
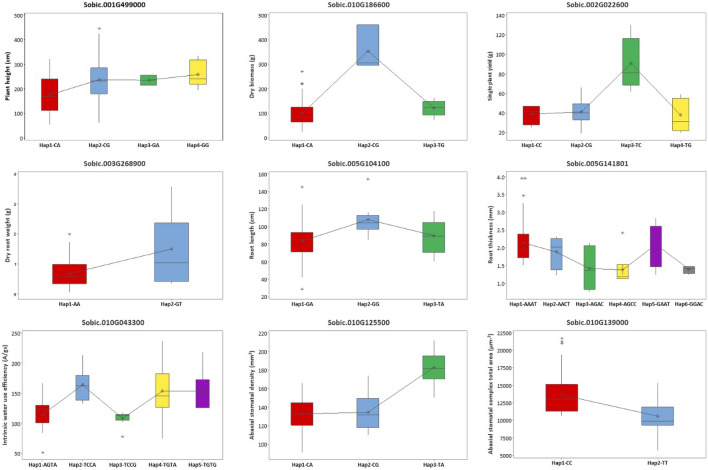
Haplotype analysis showing significant phenotypic difference between haplotypes of nine candidate genes showing significant phenotypic differences and for potential use in genomic-assisted breeding.

In this GWAS study, we identified 42 candidate genes from all evaluated traits from which five candidate genes had significant QTNs within them, while the most other candidate genes were present in proximity with the significant QTNs. Haplotype analysis was performed further for all the 42 candidate genes to validate the identified candidate genes by using non-synonymous SNPs present in the candidate genes. As this study used GBS for GWAS, only a few (2 to 4) non-synonymous SNPs with MAF > 0.05 per candidate genes were used for haplotype analysis. From the haplotype analysis, nine out of 42 candidate genes were validated showing significant difference (*p* < 0.05) between different haplotypes (Fig. 4 and Supplementary Table 3). The number of haplotype blocks formed in the candidate genes ranged from 2 to 6 showing significant difference.

## Discussion

Sorghum is resilient to biotic and abiotic stresses and its wider adaptability with low input requirements, high nutritional and food value continues to play a vital role in global food security^28^. The global demand for sorghum grain is also increasing with the US export volume steadily growing from 2.8 million tons in 2019 to about 6.6 million tons in 2020 and this is expected to increase by 2% in 2022 and more in later years (USDA, National Agricultural Statistics Service). However, productivity enhancement under stress conditions is a challenging task, because of the unpredictable nature of most periods of water and temperature stresses in sorghum growing areas and gaps in our knowledge of abiotic stress biology. In this critical situation, it is of paramount importance to focus on various biotic and abiotic stress-tolerant key traits with more emphasis of following accelerated classical and genomics-assisted breeding approaches. Uncovering QTL with significant phenotypic variation through GWAS analysis on challenging polygenic traits would help to expedite traits introgression and marker assisted selection (MAS). GWAS studies for QTL mapping use the phenotypic field data from multi-environments or controlled environment(s) to increase the mapping resolution^33–36^. Parra-Londono et al.^33^ performed GWAS using phenotypic data from one controlled greenhouse study for contrasting phosphorus availability and identified several candidate genes for root architecture traits by integrating the genotypic data in sorghum. Mandozai et al.^34^ performed GWAS in soybean on seedling root and shoot related traits and identified 27 significant SNPs with high phenotypic variance using the phenotypic data from one controlled environment study. Chen et al.^35^ conducted one greenhouse hydroponic study in soybean on recombinant inbred lines (RILs), mapped several QTL and detected *qRL16.1*, a novel QTL influencing primary root length. In cowpea, Wu, et al.^36^ performed GWAS and identified several candidate genes associated with stomatal closure from one controlled environment study.

In sorghum, several studies were attempted to explore genetic loci affecting yield and stress tolerance through GWAS^20,27–29,37^. Limited studies were conducted to dissect genomic regions responsible for root morphological and physiological traits^15,18,19,33,38^. Parra-Londono, et al.^33^ evaluated RSA under different phosphorus availability in one greenhouse environment using germination papers and bioassay plates. Similar studies have been attempted in other crops for GWAS on RSA at vegetative stage in a controlled environment^34^. The current study attempted to better utilize the germplasm resource by constructing a subset of 96 accessions and performed GWAS to co-localize the previously reported and new novel loci and candidate genes associated with agronomic, root morphological, and physiological traits.

Utilizing a large collection of germplasm in the breeding program is difficult due to various factors including linkage load of numerous undesirable genes and time consuming for characterizing various traits including physiological and biochemical traits^8^. This study initiated with the construction of a subset consisting of 96 diverse accessions representing the genetic and geographical diversity of 219 accessions which is more rewarding for enhanced utilization in breeding programs^8^. The wide coverage of genetic diversity in the constructed subset is clearly justified by the following different analyses: (i) *p*-value > 0.05 for diversity parameters of three agronomic traits performed in the subset showed no significant difference with the whole collection (Supplementary Table 4); (ii) PCA in comparison with sorghum association panel (SAP)^39^ with distinct distribution of the subset accessions (Fig. 1a); (iii) population structure analysis in the subset with a distinct separation of three main races of caudatum, durra and guinea (Fig. 1b); and (iv) LD decay to 50% r^2^ observed at 5 Kb and decay to background levels (r^2^ < 0.1) observed at ~ 80 Kb (Fig. 1c) was less than previous studies^20,29^ indicating the sufficient genomic coverage of the developed SNPs for GWAS in the subset accessions.

Several statistical methods are available to perform GWAS from which few have high accuracy and power in mapping loci. SUPER method use single locus model for mapping locus and real data has demonstrated that SUPER is superior and powerful than regular mixed linear model (MLM)^24,40,41^. FarmCPU is a multiple loci model which is robust and efficiently controls false positive and negatives where several studies have shown that FarmCPU is superior than several statistical methods for GWAS^25,42^. Method like 3VmrMLM have shown to detect all types of loci by covering quantitative trait nucleotide (QTN)-by-environment interactions (QEIs) and QTN-by-QTN interactions (QQIs) and almost unbiasedly estimate their effects, with high accuracy and power with a low false positive rate even in small mapping population^26^. In this study, GWAS was performed for all nine traits using FarmCPU, SUPER and 3VmrMLM, methods which detected 42 candidate genes together for all the traits. Haplotypes are a unique combination of jointly inherited SNPs/indels in the same chromosome segment, so haplotype analysis are a useful method for overcoming the biallelic constraints of SNPs and increasing the allelic resolution of candidate genomic areas^43^. Considering this, validation of the candidate genes identified in this study were performed by haplotype analysis which validated nine candidate genes showing significant phenotypic variance. Only the non-synonymous SNPs from candidate gene were considered for haplotype analysis as change in amino acid change in gene are caused by non-synonymous SNPs.

### Agronomic traits

This study detected QTNs and QEIs for agronomic traits, as they were evaluated for two field environments. As global climate change increases, QEIs become increasingly important in the genetic dissection of complex quantitative traits in crops^44^. Several QEIs and QTNs detected for agronomic traits were co-localized in already reported and characterized QTLs indicating the efficiency of the subset used in this study that can be used for mapping any traits of interest. Sorghum has four key dwarfing loci (*dw1*- *dw4*) that affect plant height, of which *dw1, dw2,* and *dw3* have been characterized^45–47^. In this study, a QEI (S6_44429741) was detected to be associated with plant height which was colocalized in *dw2* locus (21.2 Mb–44.99 Mb) and this QEI was found to be ~ 1.6 Mb away from the causal gene Sobic.006G067600, a protein kinase in *dw2* locus controlling height. S9_56525300, a QTN detected to be associated with dry biomass by FarmCPU was co-localized in *dw1* locus (53.89 Mb–57.86 Mb) which regulate stem internode length in sorghum. However, this QTN was ~ 513 kb away from Sobic.009G229800, the causal gene for *dw1* locus.

QEIs were detected for plant height which resulted in several novel candidate genes from which Sobic.001G499000, a potassium channel tetramerization domain containing protein present in QHGT_S4_1.1 (QTL), a reported QTL for plant height^48^ where a non-synonymous QTN (S1_76855452) was detected. Haplotype analysis was performed to validate further by using two non-synonymous SNPs in Sobic.001G499000 revealing a significant phenotypic difference in plant height between the four haplotypes formed (Fig. 4 and Supplementary Table 3). S10_52722710 was detected as a non-synonymous significant QEI associated with dry biomass and were co-localized in Sobic.010G186600, a nucleoporin-related gene present in SDI, an already reported QTL for stem morphology^49^. Nucleoporin related genes have shown to regulate nuclear transport and maintain tissue integrity mediating development of leaves in tobacco increasing the potential of this candidate gene to be associated with dry biomass^50^. This gene formed three haplotypes showing significant difference in phenotype validating highly for its association with dry biomass (Fig. 4 and Supplementary Table 3).

NGS has accelerated work in cloning genes underlying grain yield-related features over the last two decades in sorghum^51^. Previous studies have shown that qTGW1a (Sobic.001G341700) encoding G-protein γ subunit located at the N-terminus are responsible for grain weight in sorghum^52^. Multi-seeded (MSD) genes including MSD1 (Sobic.007G021140)^53^, MSD2 (Sobic.004G078600)^54^, and MSD3 (Sobic.001G407600)^55^ have reported to regulate grain number per panicle in sorghum. This study did not detect any QEIs or QTNs in proximity with the functionally characterized genes. However, some detected QTNs were co-localized in already reported QTLs increasing the potential of the candidate genes at the QTN proximity to affect plant yield. Sobic.002G022600 encoding AP2-like ethylene-responsive transcription factor AINTEGUMENTA was identified as a candidate gene which was present in proximity with S2_2070779, a significant QTN detected to be associated with plant yield by FarmCPU. This gene function has been reported to affect inflorescence branching and kernel number in maize indicating the possibility of the detected candidate gene to affect yield^56^. This candidate gene is present in qtl2bGWP, an already reported QTL^57^ for panicle weight and resulted in four haplotypes showing significant phenotypic difference validating its possibility to affect plant yield (Fig. 4 and Supplementary Table 3).

### Root morphological traits

In cereal crops, particularly in sorghum, narrow root angles have been demonstrated to play a crucial role in water-limited situations, with narrow root angles increasing availability to water from deep soil^58^. As a result, characterization, and use of selection for RSA features may hasten the development of drought-tolerant sorghum cultivars. This study detected six QTNs in total for all three root traits evaluated, from which most were co-localized in already reported QTLs. The QTNs detected to be associated with root length and root thickness were present in qRA1_5, a QTL for root angle^59^. From these QTN detected, S5_18563597 associated with root length is present near (~ 18 kb) Sobic.005G104100, peroxidase 13-related gene. Peroxidase related genes have shown to promote root hair growth at low temperature stress in *Arabidopsis* which increases the chance of the candidate gene detected to be associated with root length in this study^60^. Further validation of this candidate gene showed significant difference between three haplotypes formed (Fig. 4 and Supplementary Table 3).

### Physiological traits

Drought can be mitigated by improving water-use efficiency (WUE) at the whole plant level. Genes including *SbDof*s, *SbNAC*s, *SbWRKY30* have been found in sorghum to modulate drought tolerance in plants^61–63^. This study detected a QTN (S10_3353158) associated with iWUE in proximity (~ 213 kb) with *SbWRKY30* (Sobic.010G045700) and is present in QLpt.txs-G, a QTL affecting leaf morphology^64^. Yang, et al. ^63^ showed that *Arabidopsis* and rice plants benefit considerably from heterologous expression of *SbWRKY30* and also it directly activate the drought stress-responsive gene *SbRD19* in sorghum. The detected QTN in proximity with *SbWRKY30* from this study is a non-synonymous SNP present in Sobic.010G043300, homologous to Traes_5BL_8D494D60C, encoding inhibitor of apoptosis. Several studies have shown that anti-apoptosis genes regulate and improve stress tolerance in *Arabidopsis* and rice^65^. This candidate gene showed five haplotypes with significant difference between them further validated its potential to be associated with iWUE (Fig. 4 and Supplementary Table 4).

Genetic manipulation of pathways influencing stomatal patterning, regulation and development are shown to increase yield by optimizing water use in several crops^17^. *ERECTA*, leucine-rich repeat receptor-like kinase gene in *Arabidopsis* is known to regulate stomatal density, and patterning^66^. In rice, *OsSRO1c* plays a dual role in drought tolerance by inducing stomatal closure and H_2_O_2_ accumulation^67^. There are no reported functionally characterized genes for stomatal density (SD) and development in sorghum. This study detected a significant non-synonymous QTN (S10_15510030), co-localized in Sobic.010G125500, encoding zinc finger, C3HC4 type domain and present in LA3, a QTL responsible for drought tolerance^49^. Zinc finger protein have shown to mitigate abiotic stress tolerance via stomatal aperture control in rice which increases the chance for the detected candidate gene to be associated with SD^68^. Sobic.010G125500 showed significant difference between three haplotypes formed validating its potential to be associated with SD (Fig. 4 and Supplementary Table 3).

In summary, GWAS is performed to capture total additive genetic variance explained by heritability to understand the genetic basis of complex traits. Hence, heritability is one of the important parameters to predict gene mapping power in GWAS to some extent. In this study, the complex quantitative agronomic, root and physiological traits governed by polygenes recorded moderate (53% for root length) to high heritability (97% for plant height) indicating high additive fixable genetic effects of these traits. This study attempted to detect QEIs for agronomic traits and QTNs for agronomic, root morphological and physiological traits using three different models including FarmCPU, SUPER and 3VmrMLM. The detected 42 QTNs in this study have potential in sorghum breeding as the QTNs had high phenotypic variance (R^2^) ranging from 3 to 20%. Results from this study indicates that, 3VmrMLM is the best choice for mapping any trait of interest in particular with small mapping population. FarmCPU and SUPER did not detect any QTN associated with root and physiological traits while 3VmrMLM detected in this study. The validation of nine candidate genes associated with different traits using haplotype analysis which were co-localized with the previously reported QTL (Fig. 4) have potential for traits introgression breeding. Sobic.010G186600, a nucleoporin-related candidate gene for dry biomass and validated by haplotype analysis showing three haplotypes with significant phenotypic difference. A novel candidate gene Sobic.002G022600 encoding AP2-like ethylene-responsive transcription factor was validated showing four haplotypes with significant phenotypic difference. Several candidate genes were validated to be associated with different root and physiological traits including Sobic.005G104100, peroxidase 13-related gene with root length, Sobic.010G043300, homologous to Traes_5BL_8D494D60C, encoding inhibitor of apoptosis with iWUE, Sobic.010G125500, encoding zinc finger, C3HC4 type domain with Abaxial stomatal density are having more breeding value. In addition, the subset of 96 lines used in this study covers a vast amount of genetic diversity represented by the SAP (Fig. 1a–c) indicating its potential to map any traits of interest. The new genomic regions detected for root architecture and stomatal density identified in this study proxies for high temperature and drought tolerance. Though the candidate genes were validated through haplotype analysis to some extent, the importance and value of the identified candidate genes need to be validated strongly through linkage mapping, gene differential expression, and GO/KEGG pathway analyses. The significant QEIs and QTNs detected in this study can be used for trait(s) introgression/stacking using marker-assisted backcrossing into elite sorghum parental lines. Generating bi-parental mapping populations by crossing elite sorghum lines with accessions from the subset for desirable alleles, will enable mapping the genomic regions for validation in the future studies.

## Materials and methods

### Plant materials

A panel of 219 sorghum accessions of diverse origin (15 countries) and differing widely in their grain color was obtained from the National Bureau of Plant Genetic Resources (NBPGR), New Delhi, India (Supplementary Table 5). All 219 sorghum accessions were grown during 2019 and 2020 Rabi season (September–October to January–February) at Agricultural Research Station, Kovilpatti, India (Latitude 9.17′ N, Longitude 77.88′ E). The experimental design and field management were maintained the same for both years as described previously^69^.

### Construction of a subset/association mapping panel

Genotyping of 219 sorghum accessions using 17 SSR markers generated in our earlier study^70^ was used to perform population structure analysis through Bayesian approach in the STRUCTURE 2.3.4 tool^71^. Parameters were set with 100,000 burning period and 100,000 Markov Chain Monte Carlo (MCMC) reps after burning and K assumed population value was set from 1 to 10 with 10 iterations. Optimum K value was fixed by using STRUCTURE harvester^72^. A representative subset was constructed based on the advanced M strategy using heuristic search available with PowerCore^73^. To validate the representativeness of the constructed subset, student’s t test for Nei’s genetic distance and Shannon’s information index, and one way ANOVA and Levene’s test for variance were performed for three major agronomic traits recorded during Rabi’2019 for the entire 219 lines and the subset of 96 accessions^39,74^.

### Phenotyping of the panel

#### Agronomic traits

Data on three major agronomic traits including plant height (PH), single plant yield (SPY) and dry biomass (DB) recorded in Rabi 2019 and Rabi 2020 was used. Three plants per accession in each replication were selected for recording the data on these traits by following the sorghum descriptors^75^.

#### Root traits

Root morphological traits like root length (RL) in cm, root thickness (RT) in mm, and dry root weight (DRW) in g, were measured during the vegetative stage in all the 96 accessions grown under Greenhouse conditions at Tamil Nadu Agricultural University, Coimbatore, India (11.0152° N, 76.9326° E). Three replicates of each accession were planted in individual Polyvinyl chloride (PVC) pipes (90 × 10 cm^2^) each with one seed. PVC pipes were filled with soil mixture consisting two parts of coir pith and one part of soil at the 45^th^ day from planting for all replicates of accessions to maintain homogeneity.

#### Stomatal traits

Stomatal traits (stomatal density and stomatal size) and gas exchange parameters were measured in all the 96 accessions grown under greenhouse conditions as described above. Accessions were raised in pots with soil mixture (1:1:1 soil: coir pith: vermicompost) with two replications per entry. Gas exchange parameters viz., photosynthesis rate (A, µmol CO_2_ m^−2^ s^−1^), stomatal conductance (gs, mol H_2_O m^−2^ s^−1^) and transpiration rate (TR) (E, mmol H_2_O m^−2^ s^−1^) were measured in 45 days old plants in the second leaf from the top using LCi-SD Ultra Compact Photosynthesis System (ADC BioScientific Ltd, Hoddesdon, Herts, UK). Intrinsic water use efficiency (iWUE) was calculated by dividing photosynthesis rate by stomatal conductance (gs). All these measurements were recorded between 10.00 am and 12.00 noon (IST) with five observations at the second leaf in each replication to maintain homogeneity.

Stomatal imprints of abaxial leaf surface were taken from the second leaf from the top using nail polish and used for measuring stomatal density (SD), stomatal complex area (SCA) and stomatal complex total area (SCTA)^76^. Leaf samples were rinsed with water to remove dust particles and thin film of nail polish was applied to abaxial leaf surfaces followed by drying for 3–5 min. Imprints (~ 25 × 17 mm^2^) were taken from abaxial leaf surfaces using cellophane tape and the same were placed on the microscopic slide (75 × 25 mm^2^) and viewed at 200X magnification using 20X NIS60 planar lens (20X/0.5ph) on RXLr-5 (NEXcope) fluorescent microscope having Jenoptik optical system with ProgRes® capture camera control system (v 2.10.0.1). Stomatal images were captured in jpeg file format at the highest resolution (2580 × 1944 HQ) with microscopic view area of 0.27 mm^2^. Five microscopic images were recorded in all replications on abaxial leaf surfaces, with a total of 960 images (96 accessions x two replications x five images) to estimate SD (mm^2^), SCA (µm^-2^) and SCTA (µm^-2^). SD was calculated separately by manually counting the total number of stomata in the field of view divided by 0.27 mm^2^. SCA was calculated by ProgRes® from five randomly selected stomata of each image, SCTA was calculated by multiplying the total number of stomata by its respective SCA in each image. Frequency distribution was performed for these traits using Minitab 19^77^ and heritability was performed using metan, an R package^78^.

### Genotyping-by-sequencing (GBS) and SNP filtering

Genomic DNA was extracted from 40-day old leaves of all the 96 accessions using CTAB method^79^ and high-quality DNA was used for GBS analysis. The assessment of DNA quality and quality was done by running on 1% agarose gel. GBS libraries were prepared using the protocol adapted from Elshire, et al. ^23^. Type II restriction endonuclease (ApeKI) was used for DNA digestion and ligated to the adapter, followed by 96-plex library was constructed as per GBS protocol. Sequencing was carried out using HiSeq X sequencing platform (M/s. Oneomics Private Limited, India).

Sequence reads (FASTQ files) were processed and analyzed for SNP detection using \an in-house pipeline. Pipeline allows searching of all raw sequencing reads with perfectly matched barcode and expected remnant bp of restriction cut site and reads were further sorted, de-multiplexed and trimmed by using Trimgalore to create a unique, 150-bp long sequences called tags. These good quality tag sequences were aligned with BTx623 sorghum reference genome^80^ using Burrows-Wheeler Alignment (BWA) software^81^, while reads carrying “N” had been removed to perform further analysis. The perfectly matched and aligned sequences were processed further for SNPs calling and genotyping through freebAYES pipeline. SNPs filtering was done using TASSEL 5.0^82^, where SNPs with missing rate > 20%, minor allele frequency (MAF) < 0.05 and Heterozygosity > 0.1 were excluded, and further missing SNPs were imputed using Beagle imputation 5.0^83^. A total of 43,452 robust SNPs were retained for GWAS. Density of the filtered SNPs on the sorghum genome was visualized using CM plot, an open R package^84^.

### Population structure and linkage disequilibrium

Population structure of the subset was inferred using principal component analysis and model-based maximum likelihood approach. Linkage disequilibrium (LD) and principal component analysis (PCA) for the constructed subset was characterized and aligned with sorghum association panel (SAP)^20^. First, common SNPs with 0.01 MAF and 80% missingness from the constructed subset and 401 global sorghum accessions were filtered and retained. Next, PCA was performed where two PCA axes were built with previously published *Ape*KI GBS data of 401 global sorghum accessions^20^, and the constructed subset were projected on these axes using *prcomp* and *predict* function in R^85^. LD decay of the SAP and the subset was calculated and plotted using ~ 100 K SNPs and ~ 10 K SNPs respectively, where parameters were set for MaxDist 500 kb and 0.05 MAF. Detailed description of PCA and LD decay analyses were described in Marla, et al.^86^. Model-based maximum likelihood approach implemented by ADMIXTURE v1.23^87^ were performed for inferring population structure of the constructed subset using 4014 SNPs retained after thinning with 0.05 MAF from TASSEL 5.0.

### Genome-wide association study (GWAS)

Phenotypic data of all the agronomic, root and physiological traits was integrated against the genotyping data of 43,452 robust SNPs to perform GWAS for identifying QTNs) linked with all nine (3 agronomic, 3 root and 3 physiological) traits evaluated, and QEIs associated with three agronomic (plant height, single plant yield and dry biomass) traits. Three methods FarmCPU, SUPER, and 3VmrMLM were used to perform GWAS on all the nine traits. These three methods were performed using genome association and prediction integrated tool (GAPIT v3.0) and 3VmrMLM available in the R statistical package^24,25,41,88^. Significant QTNs exceeding Bonferroni correction threshold (5.9) from all traits were compared against known QTL for the related traits and candidate genes within/near significant SNPs (~ 50 kb) were searched using Sorghum QTL Atlas^89^ and Phytozome v.13. Haplotype analysis was performed for all the identified candidate genes using Haploview 4.2^90^ but only non-synonymous SNPs in the candidate genes were considered for haplotyping. Duncan analysis was performed to test statistical significance to understand phenotypic performance of each haplotype.

All methods in this paper were carried out in accordance with relevant guidelines in the method section.

### Supplementary Information

Supplementary Information 1.

Supplementary Information 2.

## Data Availability

The genomic data of the subset generated in this study have been deposited into the NCBI database under accession code PRJNA938246 and Mendeley public repository. https://data.mendeley.com/datasets/8yb7tnhkb4/1 (Accession 1–75) and https://data.mendeley.com/datasets/27nbpmv8rd/1 (Accession 76–96).
