# Supplementary material for: Pilot-scale genome-wide association mapping in diverse sorghum germplasms identified novel genetic loci linked to major agronomic, root and stomatal traits
Source: Sci Rep. 2023 Dec 8;13:21917. doi: 10.1038/s41598-023-48758-2 (PMC10713643; doi:10.1038/s41598-023-48758-2)
Supplement: Supplementary file 1 — Supplementary Information 1. [file 41598_2023_48758_MOESM1_ESM.docx]

**Supplementary figures**


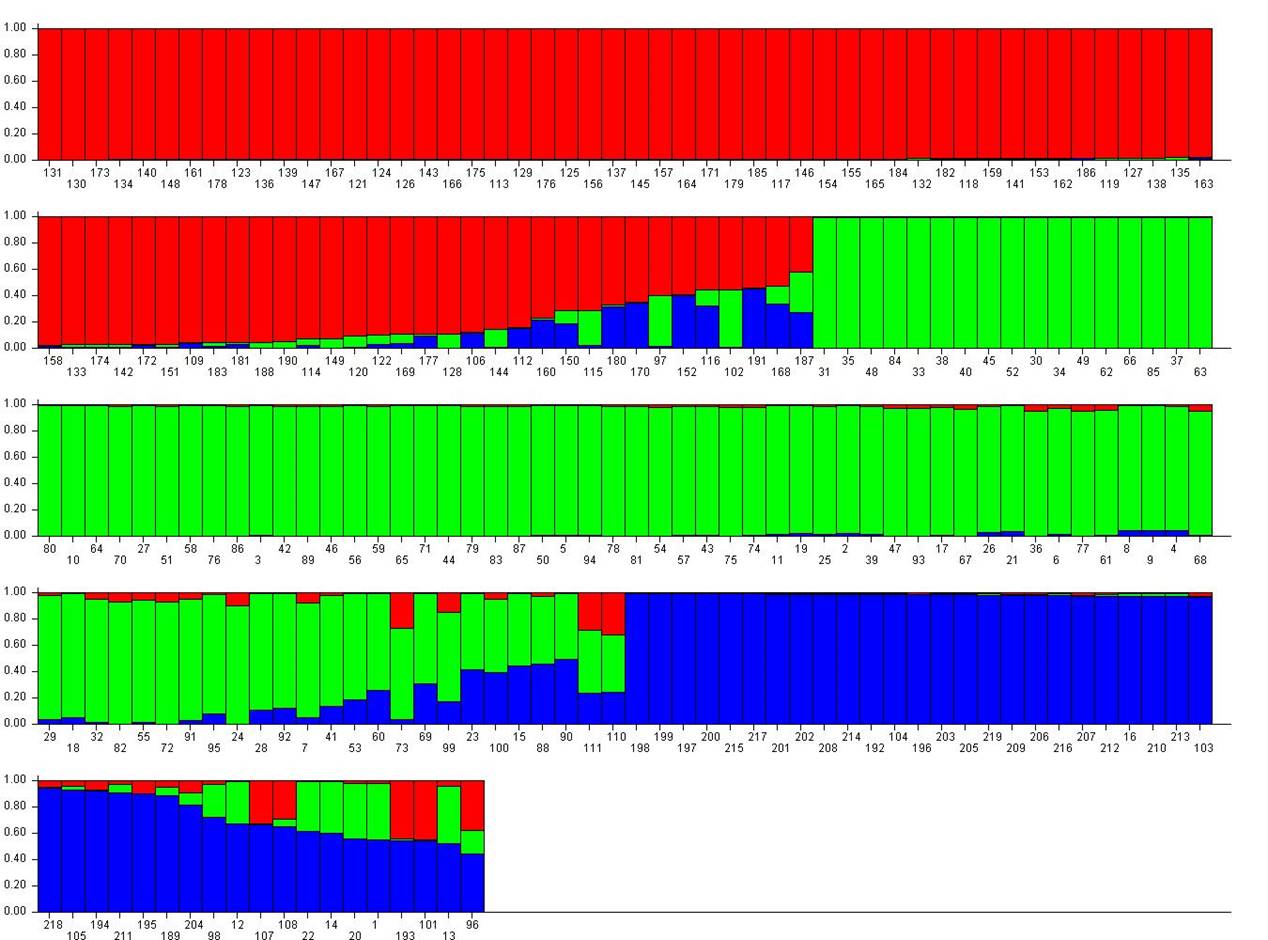


**Supplementary Figure 1.** Population structure of 219 sorghum accessions from SSR data resulting distinct grouping of accessions based on its geographical origin. Sub-population P1 (red) had most accessions from Ethiopia, Sub-population P2 (green) had most accessions from Central Africa and Sub-population P3 (blue) had most accessions from Cameroon.

**
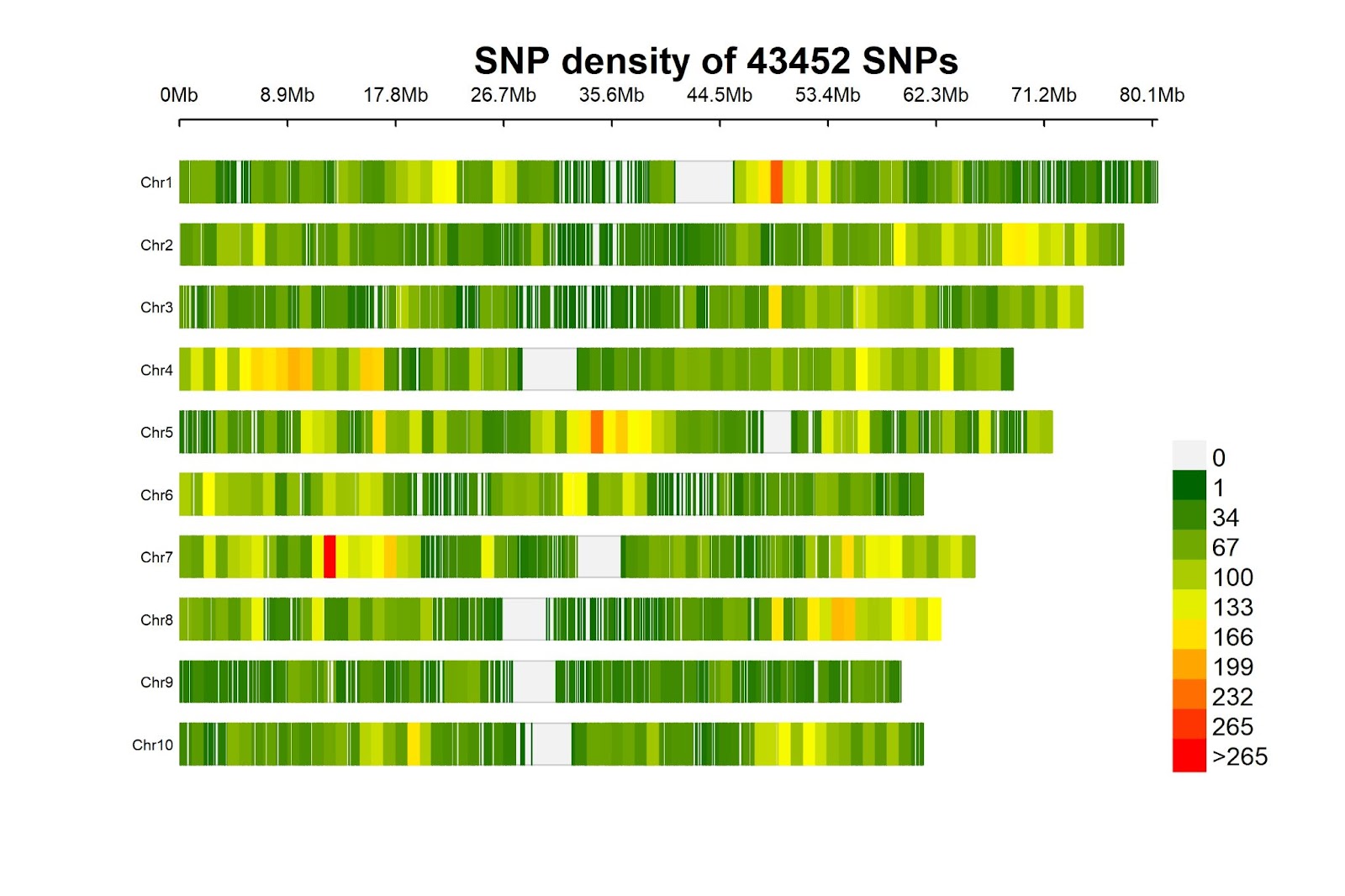
Supplementary Figure 2.** Chromosome wise distribution and density of filtered SNP set (43,452 SNPs) used in GWAS. Heatmap legend denotes the density of the SNPs present in that physical position.


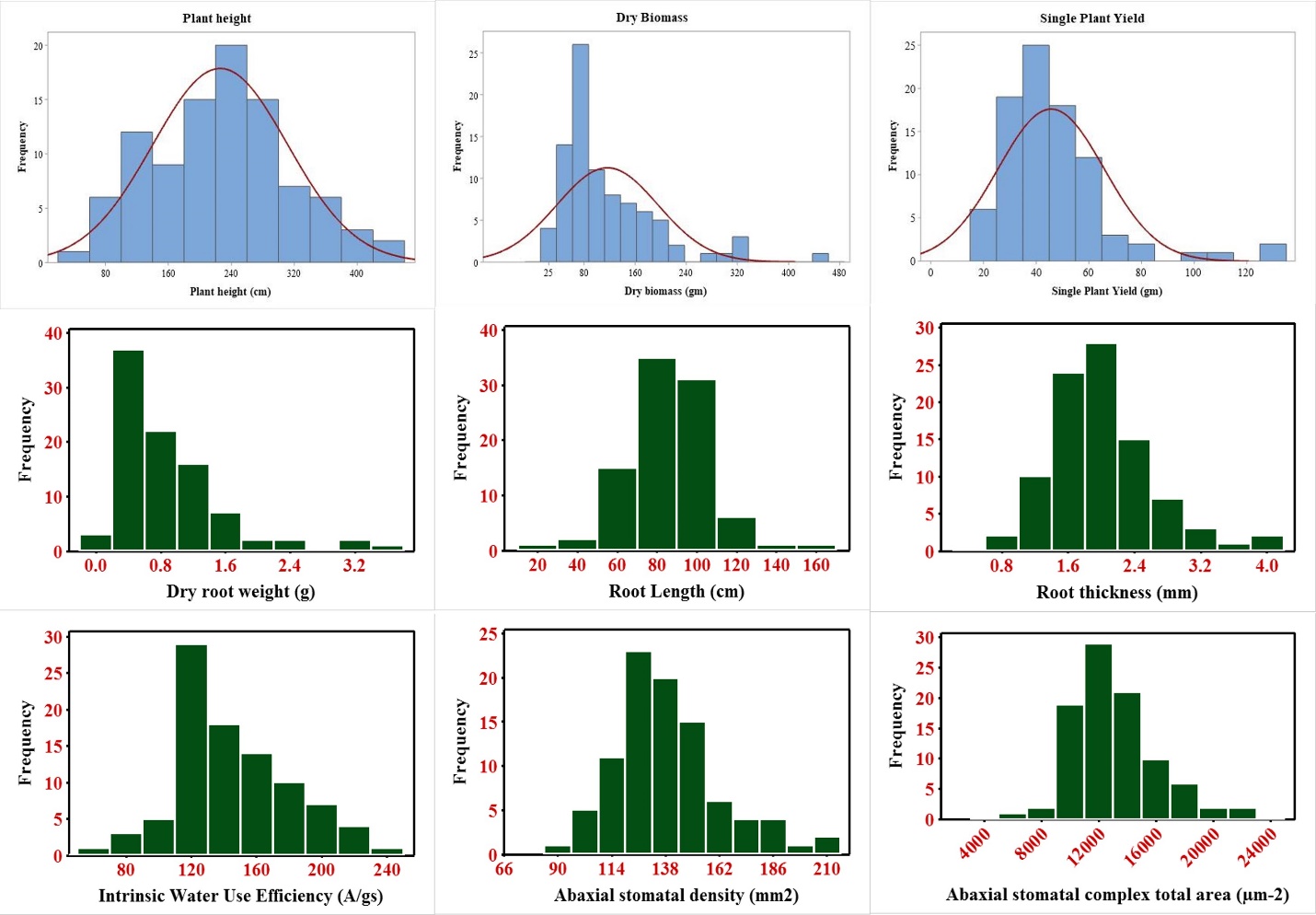


**Supplementary Figure 3**. Frequency distribution of the constructed subset showing normal distribution with continuous variation for all the traits used in GWAS.


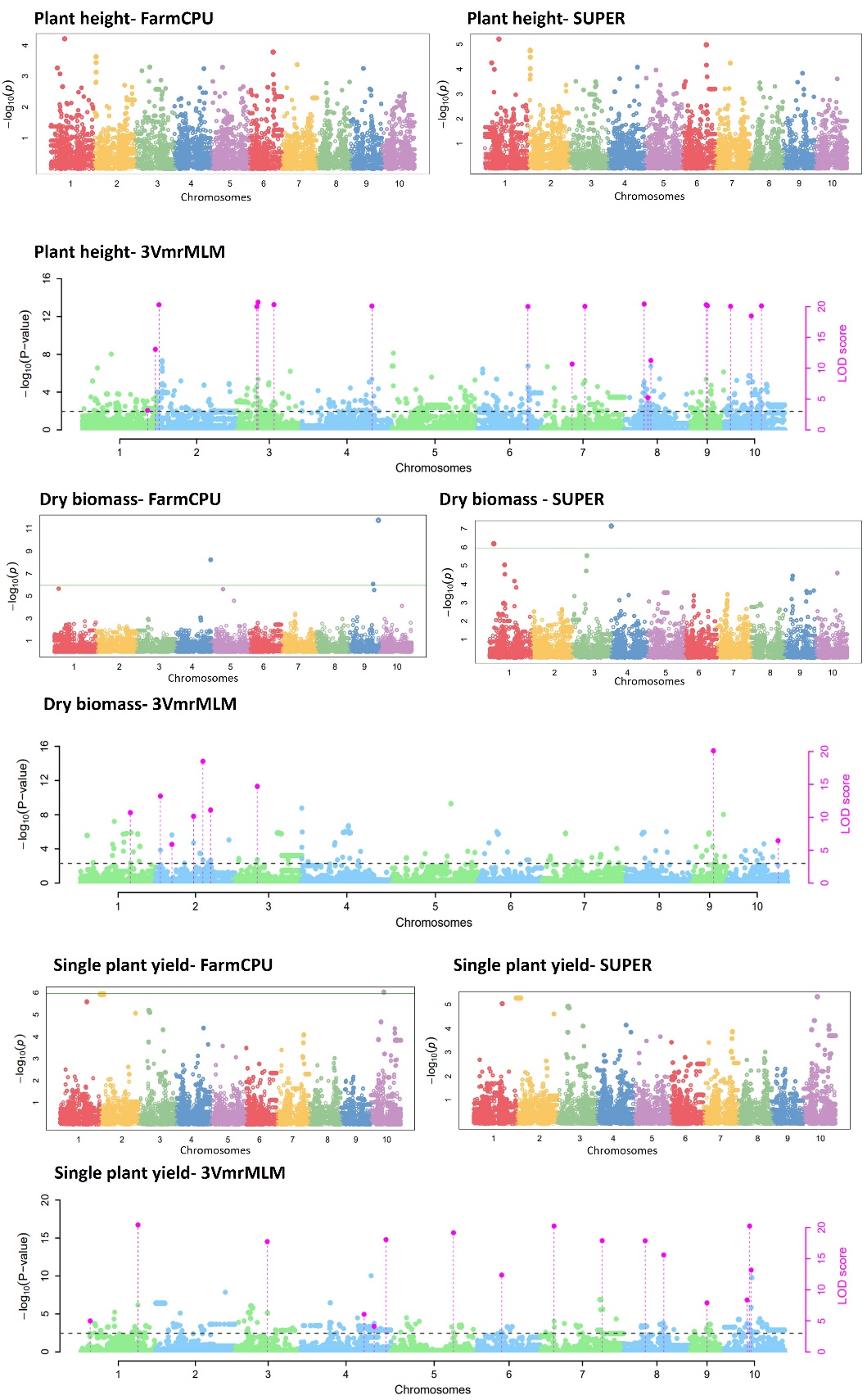


**Supplementary Figure 4**. Genome-wide association studies using FarmCPU, SUPER and 3VmrMLM methods for three agronomic traits


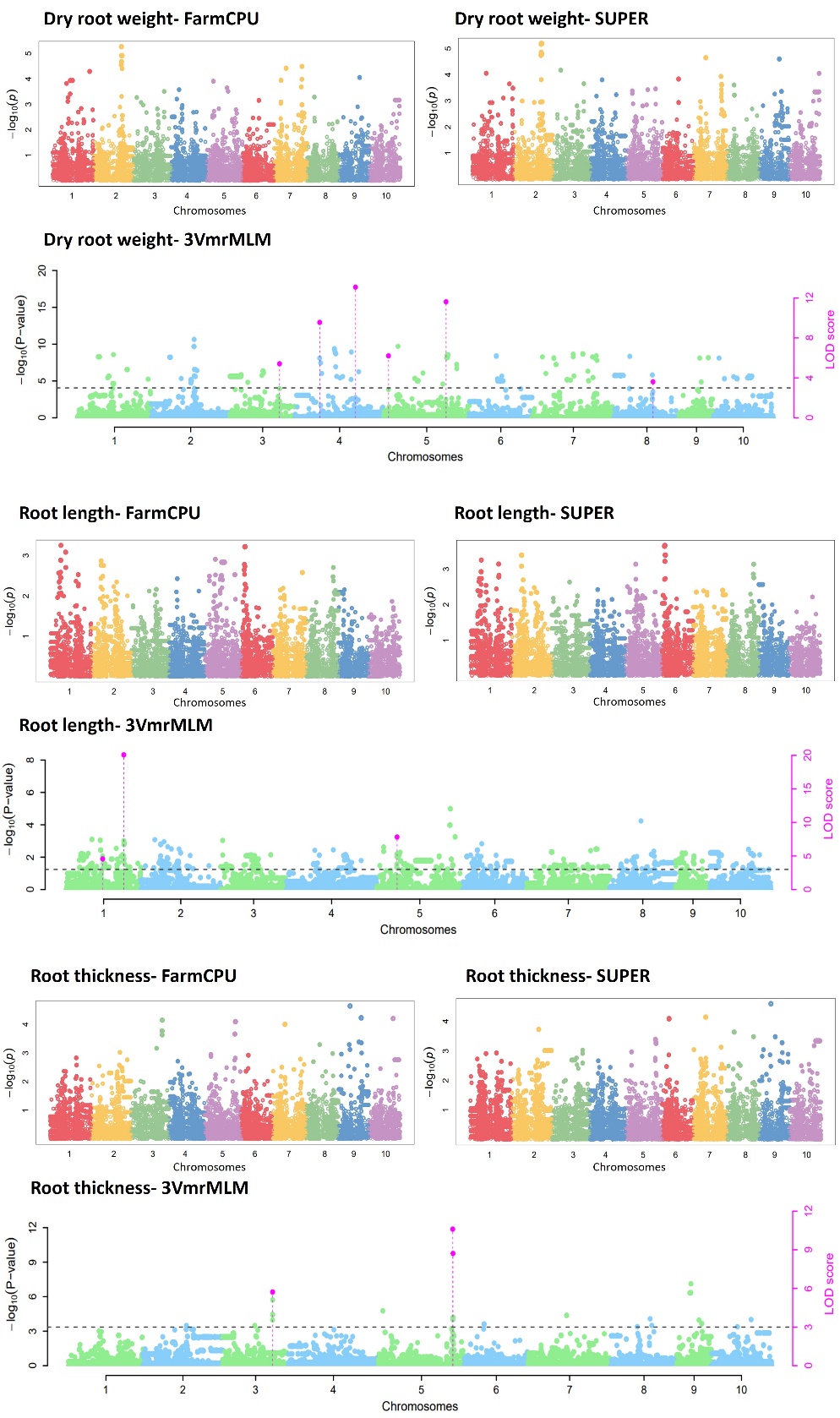


**Supplementary Figure 5**. Genome-wide association study using FarmCPU, SUPER and 3VmrMLM methods for three root morphological traits.


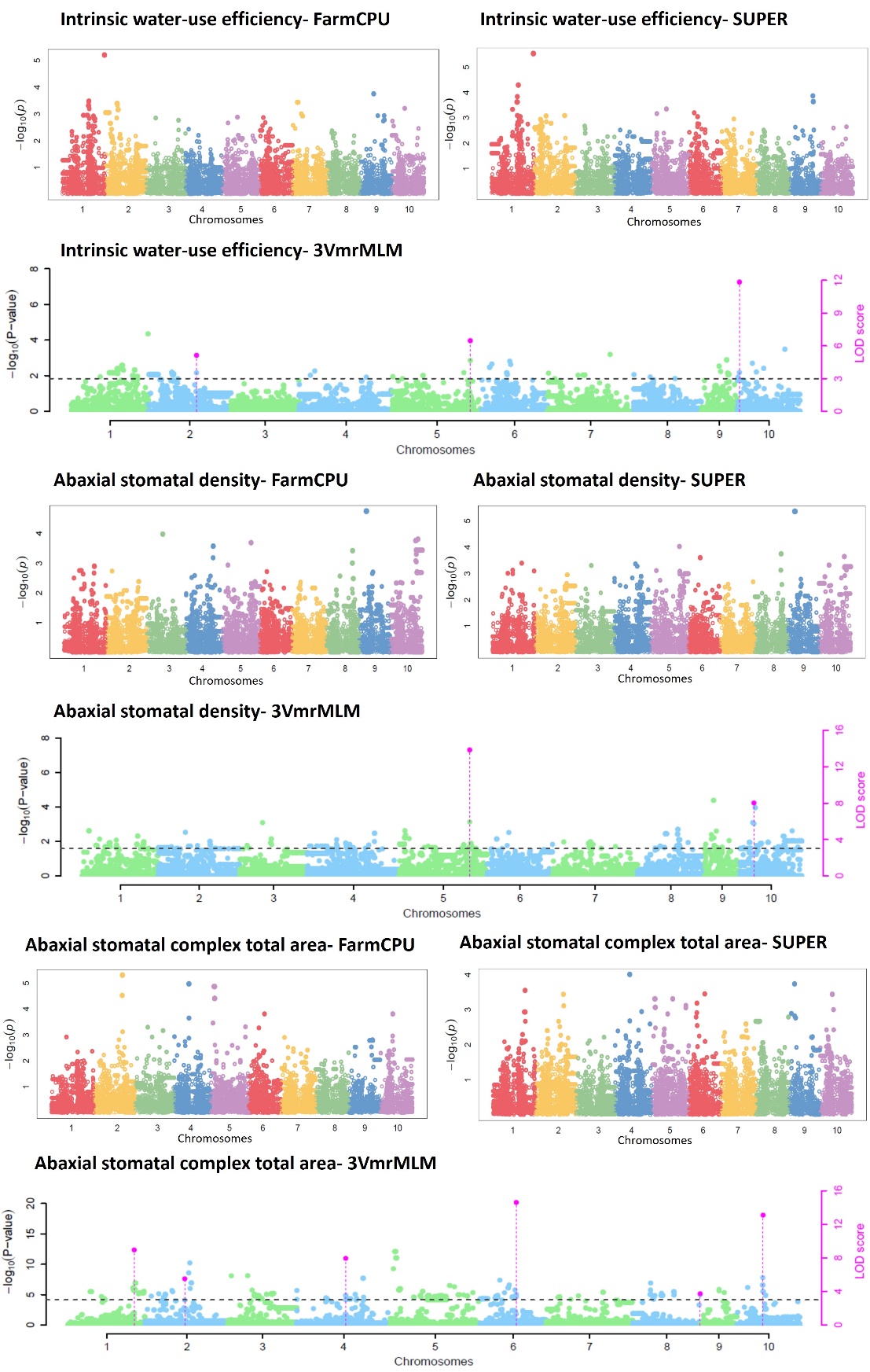


**Supplementary Figure 6.** Genome-wide association studies using FarmCPU, SUPER and 3VmrMLM methods for three physiological traits.
